# O043. Frequency-dependent habituation deficit of the nociceptive blink reflex in cluster headache and paroxysmal hemicrania

**DOI:** 10.1186/1129-2377-16-S1-A91

**Published:** 2015-09-28

**Authors:** Armando Perrotta, Maria Grazia Anastasio, Gianluca Coppola, Anna Ambrosini, Roberto De Icco, Giorgio Sandrini, Francesco Pierelli

**Affiliations:** 1 Headache Clinic, IRCCS INM Neuromed, Pozzilli, Italy; 2 Department of Medical and Surgical Sciences and Biotechnologies, “Sapienza” University of Rome Polo Pontino, Rome, Italy; 3 IRCCS Neurological National Institute C. Mondino, Pavia, Italy

## Background

The habituation phenomenon is a frequency-dependent form of non-associative learning which reflects the excitability level of both sensory and pain systems. We previously demonstrated a frequency-dependent deficit of habituation of the conventional blink reflex in cluster headache (CH)[1]. We investigated the habituation of the trigeminal nociceptive system by studying the habituation of the late component (R2) of the nociceptive blink reflex (nBR) in a wide range of stimulation frequencies in CH and paroxysmal hemicrania (PH).

## Methods

We studied 12 episodic CH patients during both, active and remission period, 12 PH patients and 20 controls. We delivered a series of 26 electrical stimuli, at different and randomly chosen stimulation frequencies (0.05, 0.1, 0.2, 0.3, 0.5, and 1Hz), subsequently subdivided into five consecutive blocks of five averaged and rectified responses for each stimulation frequency. Habituation was measured as the percentage decrease of the mean area under the curve of the R2 component across the blocks.

## Results

A significant habituation deficit of the nBR was found at higher (1Hz and 0.5Hz) and intermediate (0.5 and 0.3Hz) frequencies in CH during both active and remission phase, as well as in PH, when compared to controls. No differences in the habituation rate were found at lower (0.1 and 0.05Hz) frequencies between patients and controls.

## Conclusions

A frequency-dependent habituation deficit in trigeminal nociception was clearly detected in CH and PH, indicating a common abnormal processing of trigeminal nociception in these two different TACs. In addition, in CH this abnormal pain processing at trigeminal level is independent of the clinical activity of the pathology.

Written informed consent to publication was obtained from the patient(s).
